# Transplant oncology – Current indications and strategies to advance the field

**DOI:** 10.1016/j.jhepr.2023.100965

**Published:** 2023-11-16

**Authors:** Felix J. Krendl, Ruben Bellotti, Gonzalo Sapisochin, Benedikt Schaefer, Herbert Tilg, Stefan Scheidl, Christian Margreiter, Stefan Schneeberger, Rupert Oberhuber, Manuel Maglione

**Affiliations:** 1 Department of Visceral, Transplant and Thoracic Surgery, Center for Operative Medicine, Medical University of Innsbruck, Austria; 2 Multi-Organ Transplant Program, University Health Network, Toronto, Ontario, Canada; 3 Department of Medicine I, Gastroenterology, Hepatology and Endocrinology, Medical University of Innsbruck, Austria

**Keywords:** Liver transplantation, transplant oncology, HCC, CCA, CRLM, NELM, HEHE, hepatoblastoma

## Abstract

Liver transplantation (LT) was originally described by Starzl as a promising strategy to treat primary malignancies of the liver. Confronted with high recurrence rates, indications drifted towards non-oncologic liver diseases with LT finally evolving from a high-risk surgery to an almost routine surgical procedure. Continuously improving outcomes following LT and evolving oncological treatment strategies have driven renewed interest in transplant oncology. This is not only reflected by constant refinements to the criteria for LT in patients with HCC, but especially by efforts to expand indications to other primary and secondary liver malignancies. With new patient-centred oncological treatments on the rise and new technologies to expand the donor pool, the field has the chance to come full circle. In this review, we focus on the concept of transplant oncology, current indications, as well as technical and ethical aspects in the context of donor organs as precious resources.

**Key points**

• The concept of transplant oncology has recently attracted renewed attention.
• Liver transplantation offers a substantial survival benefit compared to alternative treatment strategies in selected patients.
• Intricate donor and recipient matching is essential to maximise transplant benefit.
• Listing criteria are evolving from static parameters to dynamic ones, emphasising tumour biology.
• New systemic treatments, including immune checkpoint inhibitors, may offer a path to liver transplant for patients previously excluded.
• Combining technical and surgical advances has the potential to ease organ scarcity.

## Introduction

Transplant oncology is not a new concept; however, it has recently attracted renewed attention.^1^^,^^2^ The term transplant oncology was first introduced in 2015^3^ and the first international consensus meeting on transplant oncology was held in 2019. This meeting resulted in the publication of the first international consensus guidelines on the topic.[4], [5], [6] Given the progress in immunotherapy and other multidisciplinary approaches driving this field, we anticipate a widening range of indications and eligibility criteria for oncologic liver transplantation (LT). Consequently, there will be a growing demand for donor organs. This raises significant questions about advancing the field further in the face of an ongoing shortage of organ donors.

Therefore, the aims of this review are to (1) revisit the concept of transplant oncology, (2) summarise current indications, (3) shed light on technical and ethical aspects in view of expanding indications and an ongoing donor organ shortage, and (4) discuss strategies which should ultimately advance the field.

## Concept of and unmet needs in transplant oncology

The concept of transplant oncology includes not only removing a cancerous organ, en bloc with clear margins, but replacing it with a new, healthy one.[7], [8], [9] More broadly speaking, transplant oncology integrates multiple specialties of transplantation and oncology, relying on the four pillars (four Es) of transplant oncology^10^^,^^11^:

(1) the evolution of multidisciplinary cancer care;
(2) the exploration of disease mechanisms;
(3) the elucidation of tumour and transplant immunology;
(4) the extension of the limits of hepatobiliary cancer surgery.

Up until today, out of all solid organ transplantations, LT is the only one which enables curative treatment of malignancies in selected patients.^7^^,^^12^ In the early phase, transplant oncology was plagued by high recurrence rates of up to 60%, leading many centres to abandon oncologic indications in favour of non-oncologic indications.^13^ Still, the Denver group noted early on that tumour stage as well as tumour type play an important role in selecting patients for whom favourable outcomes can be achieved.^14^ These observations were later solidified in Tokyo and Pittsburgh when Yamamoto *et al.*^15^ and Iwatsuki *et al.*^12^ compared outcomes for hepatic resection *vs.* LT in patients with HCC. In patients with early and intermediate stage HCC, oncologic outcomes were satisfactory while outcomes for advanced stage HCC were poor.^12^ Similar observations with regards to the effect of tumour stage on oncologic outcomes following LT for secondary liver malignancies were made in Vienna by Mühlbacher *et al.*^16^ Limiting LT for colorectal liver metastases (CRLMs) to patients with histologically and genetically negative lymph nodes (pN0) of the primary tumour resulted in improved outcomes and sometimes even long-term survival.^17^ Still, LT for CRLMs was abandoned in the early 1990s due to high recurrence rates.^17^^,^^18^

**Fig. 1:**
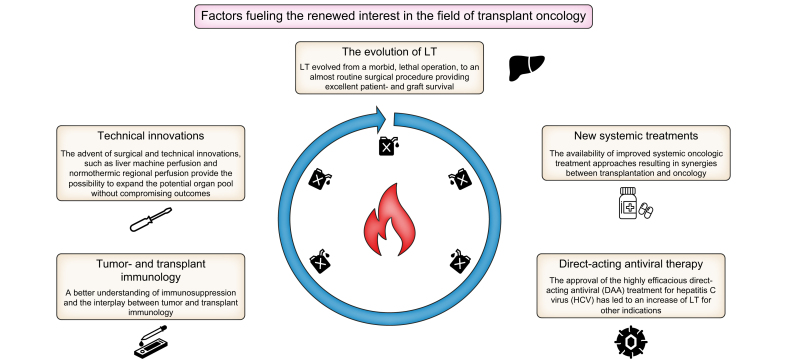
Factors fuelling the renewed interest in the field of transplant oncology.

The field of LT has always been troubled by an ever-present discrepancy between organ supply and demand. Thus, for transplant oncology to succeed, better selection criteria for oncologic indications were needed. This issue was addressed in a prospective landmark study published by Mazzaferro *et al.* in 1996. In this seminal paper the so-called “Milan criteria” were defined based on the number and size of tumour lesions to select patients with HCC for LT.^19^ Ever since, the Milan criteria have been considered the gold standard to select patients with HCC for LT and have been incorporated into regional and national allocation policies.^20^^,^^21^ While adherence to the Milan criteria has led to very good outcomes,^22^ many experts felt that the selection criteria were too restrictive, denying patients who might benefit from LT access to this lifesaving procedure.^23^ In recent years, a more thorough understanding regarding the importance of tumour biology has emerged. This has led to the development of new patient selection criteria with the focus shifting from static, morphologic selection criteria towards more dynamic criteria, which better allow for the assessment of a tumour’s biologic behaviour over time.[24], [25], [26], [27] With the important role of tumour biology in the setting of transplant oncology more clearly defined, interest in oncologic indications other than HCC such as cholangiocarcinoma (CCA), neuroendocrine tumours with liver metastases (NETLMs)^5^ and CRLMs has resurfaced.^28^ Factors fuelling the renewed interest in transplant oncology are presented in Fig. 1. Excessive expansion of inclusion criteria will result in a significant increase in organ demand, with a consequent increase in waiting time potentially jeopardising overall survival (OS) among all waitlisted patients including those with hepatic malignancies.^29^ Acknowledging that tumour biology rather than overall tumour burden dictates the disease course, will move selection criteria into focus. Furthermore, an ethical framework within the setting of transplant oncology needs to be established, since patients with primary and secondary malignancies of the liver have to compete among patients with non-oncologic liver diseases for a limited supply of organs.^30^ Patients with oncologic indications for LT usually have preserved liver function and thus low laboratory model for end-stage liver disease (MELD) scores. Consequently, these patients are disadvantaged in laboratory MELD-based allocation systems. Acceptable outcomes will need to be defined for all patients with hepatic malignancies waiting for LT, as has been done for HCC where patients fulfilling specific pretransplant criteria receive additional (exceptional) MELD points. In the past, an arbitrary 5-year OS rate of more than 50% has been postulated to be an acceptable outcome following LT.[31], [32], [33] More recently, in the US, a 5-year OS rate of 60% was suggested to be a sensible outcome in the setting of LT for HCC. Markov model analysis showed that if the 5-year OS rate lies below the 60% cut-off, the harm caused to other patients on the waiting list outweighs the benefits for the recipient.^34^ However, these results need to be viewed cautiously as the cut-off may vary from region to region depending on the availability of organs.

In summary, the following unmet needs in the field of transplant oncology can be delineated: (1) refinement of selection criteria through a focus on dynamic criteria reflecting tumour biology; (2) expansion of the donor pool, via split liver transplantation (SLT), live donor liver transplantation (LDLT) and the utilisation of machine perfusion for organ reconditioning and repair affording more patients access to LT; (3) definition of biomarkers to guide tumour tailored, oncologic and immunosuppressive treatment.

## Oncologic indications for LT – current state of affairs

### Hepatocellular carcinoma

In Western societies, HCC treatment is based on the Barcelona Clinic Liver Cancer (BCLC) algorithm, which has recently been updated for the seventh time.^35^ In the East, the BCLC algorithm finds no clinical application. Instead, the Hong Kong Liver Cancer classification is one of the accepted algorithms used to guide treatment for HCC.^36^ For HCCs occurring in cirrhotic livers LT offers the benefit of full local tumour control along with the replacement of the diseased, pre-cancerous, cirrhotic liver. Introduced in 1996 by Mazzaferro *et al.*^19^ the Milan criteria (Table 1) remain the cornerstone for selecting patients with HCC for LT in many Western countries.^37^ Looking for a way to give more patients a chance to benefit from LT, efforts have been made to widen the selection criteria while maintaining acceptable outcomes.^38^ Most notably, the University of California - San Francisco (UCSF) criteria published in 2001,^39^ which were followed by the Up-to-7 criteria in 2009^40^ (Table 1). In 2018, the Metroticket 2.0 criteria, a refinement of the Up-to-7 criteria, not only relying on imaging criteria but also including alpha-fetoprotein (AFP) levels were presented, moving away from purely morphological selection criteria.^41^ The more recently published NYCA (New York/California) score goes one step further. It not only incorporates absolute AFP levels but AFP response over time, allowing for a dynamic assessment of tumour biology rather than just focusing on one absolute value.^24^ For patients with advanced disease who initially do not qualify for LT, typically those presenting “outside Milan” or those with very high AFP levels (>1,000 ng/ml), loco-regional therapies, such as transarterial chemoembolization and radiofrequency ablation, are available as part of downstaging protocols.^42^ While universal entry criteria have not been established, most downstaging protocols use the Milan criteria as an endpoint.^43^ For patients initially outside Milan but within UCSF downstaging entry criteria (Table 2), the 5-year OS rate following successful downstaging to within Milan is similar to that in patients who have always fulfilled the Milan criteria.^44^^,^^45^ This is in line with the observation that response to downstaging allows for the accurate selection of patients with favourable tumour biology.^46^^,^^47^ Due to the favourable LT outcomes reported following successful downstaging, this strategy has been added to the recently updated BCLC treatment algorithm.^35^^,^^48^ To filter and select patients most likely to benefit from downstaging while at the same time preventing patients at high risk of dropout from entering downstaging protocols, downstaging entry criteria have been defined (Table 2).[49], [50], [51], [52], [53] Yet, since applying downstaging entry criteria will inevitably lead to the exclusion of patients who might benefit from downstaging some suggest offering downstaging to all patients outside of existing transplant criteria (all-comers) as long as no extrahepatic disease and no macrovascular invasion are present. These recommendations are based on the results of the randomised-controlled XXL trial.^54^ Despite all efforts to design optimal selection criteria, 8% to 20% of patients will develop recurrence.^55^^,^^56^ The RETREAT score, integrating explant tumour burden (diameter of largest viable tumour + number of tumours on explant pathology), presence of microvascular invasion and AFP level at transplantation (Table 3), allows for the accurate prediction of HCC recurrence.^57^^,^^58^ A patients’ RETREAT score may range from 0 to 8 points. For patients with a RETREAT score between 0 and 3 points, the 1- and 5-year recurrence risk lies below 10% and 20%, respectively.^57^ In contrast, a RETREAT score ≥5 points carries a 1- and 5-year recurrence risk close to 40% and 75%, respectively.^57^^,^^59^ While not helpful for patient selection, as it only becomes available after LT, the RETREAT score may help to guide postoperative surveillance as well as to select recipients for adjuvant therapy. At UCSF, surveillance intervals are guided by the recipient’s RETREAT score. Furthermore, patients with a RETREAT score of 4 points or more should be encouraged to enrol into clinical trials testing adjuvant therapies due to their high recurrence risk.^58^ Several studies have shown that, even if recurrence does occur, aggressive resection with curative intent of intra- and extrahepatic tumours may lead to prolonged survival.^55^^,^[60], [61], [62]

**Table 1:** Commonly used selection criteria for LT in patients with HCC listed chronologically.

Criteria	Definition	Outcome
Milan criteria^19^ Milan, Italy 1996	Single lesion ≤5 cm Up to 3 lesions, all ≤3 cm No evidence of gross vascular invasion No regional LN or extrahepatic metastases	4-year OS: 75.0%
UCSF criteria^39^ San Francisco, USA 2001	Single lesion ≤6.5 cm 2-3 lesions with largest lesion ≤4.5 cm Total tumour diameter ≤8 cm No evidence of gross vascular invasion	5-year OS: 75.2%
Shanghai Fudan criteria^227^ Shanghai, China 2006	Solitary lesion ≤9 cm in diameter ≤3 lesions, the largest ≤5 cm Total tumour diameter ≤9 cm No macrovascular invasion	3-year OS 80.0%^227^ 5-year OS 78.1%^228^
Kyoto criteria^229^ Kyoto, Japan 2007	Number of lesions ≤10 Diameter ≤5 cm PIVAK-II ≤400 mAU/ml	5-year recurrence rate 4.9% 5-year OS: 86.7%
Fukuoka criteria^230^ Fukuoka, Japan 2007	Tumour size and number not limited No gross vascular invasion No extrahepatic disease	3-year OS 68.6%
Tokyo criteria^231^ Tokyo, Japan 2008	Up to 5 nodules Maximum diameter ≤5 cm	5-year RFS: 90% 5-year OS 75.0%
Total tumour volume^232^ Multicentre, North America 2008	TTV ≤115 cm^3^	5-year OS: *Radiology:* 76.0% *Pathology:* 79.0%
Hangzhou criteria^233^ Hangzhou, China 2008	Total tumour diameter ≤8 cm Total tumour diameter > 8 cm *plus* Histopathologic grade I or II *and* Preoperative AFP level ≤400 ng/ml	5-year OS: 70.7%
Asan criteria^234^ Seoul, Korea 2008	Largest tumour diameter ≤5 cm Number of HCC lesions ≤6 No gross vascular invasion	5-year OS: 76.3%
Up-to-7 criteria^40^ Milan, Italy 2009	Largest tumour diameter (cm) + number of tumours ≤7 No vascular invasion	5-year OS: 71.2%
Extended Toronto Criteria^235^ Toronto, Canada 2011	No systemic cancer-related symptoms No extrahepatic disease No vascular invasion Tumour not poorly differentiated (Milan-out tumours only)	5-year OS: 72.0%
French AFP model^236^ Multicentre, France 2012	Low risk: ≤2 points High risk: >2 points Largest diameter (points): ≤3 cm (0), 3–6 cm (1), >6 cm (4) Number of nodules (points): 1–3 (0), ≥4 (2) AFP level (points): ≤100 ng/ml (0), 100–1,000 ng/ml (2), >1,000 (3)	5-year OS Low risk: 67.8% High risk: 47.5%
Chengdu criteria^237^ Chengdu, China 2013	Total tumour diameter ≤9 cm No macrovascular invasion	5-year OS: 79.4%
Metroticket 2.0^41^ Milan/Shanghai, Italy/China 2018	Number of lesions + largest lesion size (cm) ≤7 and AFP <200 ng/ml or Number of lesions + largest lesion size (cm) ≤5 and AFP <400 ng/ml or Number of lesions + largest lesion size (cm) ≤4 and AFP <1,000 ng/ml	5-year OS: 79.7%
NYCA score^24^ New York/Los Angeles, USA 2018	Low risk: 0-2 points Acceptable risk: 3-6 points High risk: ≥7 points Maximum tumour size (points): 0-3 cm (0), 4-6 cm (2), >6 cm (4) Maximum tumour number (points): 1 (0), 2-3 (2), >3 (4) AFP response (points) AFP always <200 ng/ml (0) Responders Max >200–1,000 ng/ml to Final <200 ng/ml (2) Max >1,000 ng/ml to Final <1,000 ng/ml (50%)∗ (2) Non-responders Max >200–400 ng/ml to Final >200 ng/ml (3) Max >400–1,000 ng/ml to Final >200 ng/ml (4) Max >1,000 ng/ml to Final >1,000 ng/ml (6)	5-year OS: Low risk 75% Acceptable risk 62% High risk 40%
5-5-500 rule^194^ Multicentre, Japan 2019	Tumour size ≤5 cm in diameter Tumour number ≤5 AFP value ≤500 ng/ml	5-year recurrence rate: 7.3% 5-year OS: 75.8%

AFP, alpha-fetoprotein; LN, lymph node; OS, overall survival.

∗ Must be a >50% drop.

**Table 2:** Downstaging entry criteria.

UNOS/UCSF^45^	Bologna^238^
One lesion > 5 cm and ≤8 cm	One lesion ≤6 cm
Two to three lesions, each ≤5 cm	Two lesions, each ≤5 cm
Four to five lesions, each ≤3 cm	Three to five lesions, each ≤4 cm
Total tumour diameter ≤8 cm	Total tumour diameter ≤12 cm
Absence of vascular invasion∗	Absence of macrovascular or biliary invasion
Minimal observation period of 3 months between completion of downstaging and listing	Minimum follow-up of 3 months during which AFP has to remain <400 ng/ml

Modified from Yao *et al.*^45^ and Ravaioli *et al.*^238^

AFP, alpha-fetoprotein; UCSF, University of California - San Francisco; UNOS, United Network for Organ Sharing.

∗ Based on *cross-sectional* imaging.

**Table 3:** RETREAT score criteria.

Predictor	Points
AFP levels, ng/ml	
0–20	0
21–99	1
100–999	2
≥1,000	3
Microvascular invasion	
Absent	0
Present	2
Largest viable tumour diameter (cm) + number of viable tumours	
0∗	0
1.1–4.9	1
5.0–9.9	2
≥10	3

Modified from van Hooff *et al.*^59^

AFP, alpha-fetoprotein.

∗ No viable tumour on explant pathology.

### Cholangiocarcinoma

Cholangiocarcinomas (CCA) amenable to LT are either adenocarcinomas affecting intrahepatic (iCCA), or perihilar (pCCA) areas of the biliary tree.

#### pCCA

Together with surgical resection, LT represents a potential curative treatment approach for pCCA. Unresectable pCCA is a well-established indication for LT within the Mayo Clinic protocol.^63^ Since their first experience, multiple modifications to the multimodal neoadjuvant protocol have been applied (Table 4) resulting in 5-year OS rates of 76% and 58% for primary sclerosing cholangitis-associated pCCA and *de novo* pCCA, respectively.^64^^,^^65^ Similarly encouraging results have been reproduced by other groups in North America and Europe, implementing the same neoadjuvant protocol pioneered at the Mayo clinic,^66^^,^^67^ and were recently confirmed in a meta-analysis.^68^ Ongoing prospective trials (NCT04378023, NCT04993131) will gather more evidence in this field.

**Table 4:** Mayo Clinic protocol with modifications.

Diagnostic- and exclusion criteria	Pretransplant treatment	Staging	Peri- and post-transplant considerations
**Diagnostic criteria** Malignant appearing stricture on cholangiography and at least one or more of the following: • Positive brush cytology or histology • Positive fluorescence *in situ* hybridisation (FISH) test • Elevated CA19-9 >100 U/ml in the absence of cholangitis • Mass seen on cross-sectional imaging **Exclusion criteria** • Radial tumour diameter ≥3 cm • Tumour that extends below the cystic duct • Prior exploration with violation of the tumour plane • Metastatic disease • A positive transperitoneal biopsy of the tumour (due to a high rate of tumour seeding along the biopsy track)	**Radiochemotherapy** • A total of 45 Gy (two daily fractions) over 2 weeks • Continuous 5-FU infusion given over the course of the EBRT **Brachytherapy** • 1 week following completion of percutaneous EBRT • 9.3 to 16 Gy (instead of 20-30 Gy in the initial protocol) using Iridium-192 **Chemotherapy** • Maintenance oral capecitabine • 2,000 mg/m^2^ per day in two divided doses, 2 out of every 3 weeks	• Chest and abdomen contrast-enhanced computed tomography • Cholangiography (percutaneous or endoscopic) • Endoscopic ultrasound guided aspiration of the regional hepatic lymph nodes prior to neoadjuvant therapy - patients with LN metastases are excluded **Hand-assisted staging laparoscopy** • Includes a complete exploration of the abdominal cavity with routine biopsy of regional LN as well as an evaluation of the caudate lobe (if LDLT is planned) • The LN overlying the common hepatic artery at the take-off of the GDA and one LN along the distal common bile duct (CBD) in addition to any suspicious LNs should be sampled • Seprafilm® (Sanofi-Aventis) is applied to prevent adhesions for patients that stage negative and are awaiting a DDLT • For DDLT the staging operation is performed close to the expected transplant date • For LDLT the staging operation is performed one day prior to LT	**Piggy-back** • Caval-sparing hepatectomy can be performed if the caudate lobe thickness permits an adequate resection margin **Arterial jump graft** • Due to radiation induced hepatic artery injury reconstruction with a donor iliac artery interposition graft to the infrarenal aorta is recommended for all patients irrespective of the hepatic artery appearance • For LDLT the Mayo group reverted to using the native recipient common hepatic artery along (due to the large size mismatch) with close observation and early intervention **Portal vein interposition graft** • The portal vein and CBD are divided as close to the pancreas as possible resulting in a short recipient portal vein • For LDLT a deceased donor iliac vein is often necessary as an interposition graft **Biliary reconstruction** • Malignancy clearance requires low division of the CBD which precludes a duct-to-duct anastomosis *(i.e*., a choledocho-jejunostomy is required in all cases) **Anticoagulation** • Anticoagulation is started as soon as INR is below 2 • Patients are maintained on aspirin indefinitely

CA-19-9, carcinogenic antigen 19-9; DDLT, deceased donor liver transplantation; EBRT, external beam radiotherapy; FISH, fluorescent *in situ* hybridisation; FU, fluorouracil; INR, international normalized ratio; LDLT, live donor liver transplantation; LN, lymph node; LT, liver transplantation.

Fuelled by the promising outcomes following LT some argue that patients suffering from resectable pCCA might also benefit from LT following neoadjuvant multimodal treatment. A recent multicentric retrospective analysis reports 5-year OS rates of 54% in transplanted lymph node-negative patients with pCCAs <3 cm (excluding primary sclerosing cholangitis-associated tumours) compared to 29% in resected patients.^69^ However, due to the unusually poor outcomes reported following these resections of early stage pCCA these findings need to be viewed cautiously. Hence, at present, liver resection remains the recommended treatment of choice for resectable *de novo* pCCA.^70^ A prospective, randomised-controlled, multicentre study in France (TRANSPHIL, NCT02232932) expected to shed more light on this topic was prematurely terminated due to accrual issues.

#### iCCA

Since most patients present with large, advanced stage tumours, only approximately one-fourth are eligible for resection,^71^ and 5-year OS rates range between 25% and 40%.^72^ Given the high incidence of iCCA in Asia, the increasing incidence of iCCA in high-income Western countries^73^ and its dismal prognosis following resection, alternative treatment strategies are urgently needed. iCCA as a formal contraindication for LT has been challenged by a retrospective Spanish multicentre study. Patients with incidentally found single tumours ≤2 cm, without any vascular or biliary involvement or extrahepatic manifestation, defined as “very early” iCCA attained a 5-year actuarial survival rate of 73%.^74^ These findings were confirmed in two other retrospective multicentre follow-up studies where comparison of incidentally found “very early” *vs.* advanced iCCA and pT1 *vs.* pT2-T4 resulted in significantly different 5-year survival rates (65% *vs.* 45%, *p* = 0.02 and 80% *vs.* 31%, *p* = 0.018).^75^^,^^76^ In line with these observations, the recently published EASL-ILCA Clinical Practice Guidelines on the management of iCCA propose the inclusion of LT as a potential treatment option for patients with very early iCCA in cirrhotic livers.^77^

More recently, the Methodist–MD Anderson Joint Cholangiocarcinoma Collaborative Committee has gathered experience in LT for locally advanced iCCAs not amenable to resection due to underlying liver disease or unfavourable localisation.^78^^,^^79^ In their prospective case series, they included patients with multifocal iCCAs >2 cm, without vascular invasion or extrahepatic spread. All patients received neoadjuvant chemotherapy and had to achieve a minimum of 6-months’ radiographic response or stability. Negative staging laparotomy and frozen sections of hilar lymph nodes were prerequisites to proceed with LT. Of 32 initially enlisted patients, 18 underwent LT. The median tumour number was two, the median tumour diameter was 10.4 cm. In this highly selected patient group, the 5-year OS rate was 57%, while recurrence occurred in 38.8% of cases.^79^ Currently three prospective clinical trials are investigating the role of LT in patients with iCCA (NCT02878473, NCT04195503, NCT04556214).

### Hepatoblastoma

Hepatoblastoma is the most common primary liver malignancy in the paediatric population with an incidence of 1.2–1.5 per million.^80^ The incidence of hepatoblastoma has been increasing,^81^ partly due to the improved survival of premature infants.^82^ While most cases are sporadic, known risk factors include Beckwith-Wiedemann syndrome, Glycogen storage diseases 1-4, familial adenomatous polyposis, trisomy 18, premature birth, low birth weight and maternal tobacco exposure.^80^^,^^81^^,^^83^ Hepatoblastomas may be of epithelial or mixed epithelial-mesenchymal origin.^84^ The clinical presentation is often unspecific. An abdominal mass may be palpated on physical examination.^80^ A definitive diagnosis requires the presence of characteristic features on cross-sectional imaging in combination with elevated AFP levels. Tissue biopsies are often obtained to confirm the diagnosis and guide management but histologic confirmation is not mandatory outside of clinical studies.^80^ Introduced in 1992 by the Société Internationale d’Oncologie Pédiatrique (SIOPEL),^85^ the pretreatment extent of disease (PRETEXT) staging system has been adopted universally to classify the overall tumour burden and guide initial treatment.^84^ Treatment response following systemic treatment is assessed via the post-treatment extent of disease (POST-TEXT) staging system.^84^ Today systemic treatment usually consists of cisplatin-based chemotherapy regimens which have resulted in improved resectability rates of up to 85%.^82^ Consequently, surgical resection in combination with chemotherapy is the mainstay of treatment for patients with low-risk, resectable hepatoblastomas (PRETEXT I or II).^86^ In contrast, patients with high-risk, borderline resectable tumours (PRETEXT III) or unresectable tumours (PRETEXT IV, PRETEXT III with macrovascular invasion) should be referred to transplant centres and evaluated for LT.^5^^,^^87^^,^^88^ All patients with PRETEXT III and IV tumours should undergo neoadjuvant chemotherapy followed by assessment of treatment response via the POST-TEXT classification system after two chemotherapy cycles.^84^ Additionally, the AFP response should be evaluated as decreasing AFP levels in response to neoadjuvant chemotherapy have been shown to predict favourable outcomes,^86^ while poor AFP response is associated with tumour recurrence.^89^ Clear indications for LT are centrally located POST-TEXT III tumours, POST-TEXT III tumours with macrovascular involvement and POST-TEXT IV tumours.^5^^,^^82^ The presence of extrahepatic metastases is no contraindication to LT if the metastases are chemo-responsive or amenable to surgical resection.^5^ Five-year OS rates following LT for hepatoblastoma have significantly improved over time and are approaching 90% in the current era of transplant oncology.^82^^,^^89^

Salvage LT (LT performed for incomplete resection or tumour recurrence following prior liver resection) has historically been associated with poor outcomes and its use has thus been controversial.^87^ More recent studies, however, paint a different picture as survival rates following salvage LT were similar to those reported for primary LT,^86^^,^^90^^,^^91^ positioning salvage LT as a potential lifesaving option among selected patients.

### Hepatic epithelioid hemangioendothelioma

Hepatic epithelioid hemangioendothelioma (HEHE) is a rare vascular tumour with varying malignant potential.^92^^,^^93^ Its clinical course may range from benign to highly malignant, making its prognosis unpredictable.^92^^,^^94^ Standard radio- and chemotherapy are largely ineffective, and surgical treatment is the only curative option.^92^^,^^95^ Liver resection and LT are two common surgical options, with no significant difference in OS between the two procedures for resectable disease with favourable prognostic factors.^96^^,^^97^ However, since HEHE commonly shows a multifocal pattern, affecting both liver lobes in over 80% of cases, LT is often the only chance for cure.^98^ Retrospective studies show that LT can offer good 5-year OS rates, ranging from 64% to 83%.[98], [99], [100], [101] Based on these favourable results, UNOS (United Network for Organ Sharing) now grants MELD exception points to patients with biopsy-proven unresectable HEHE irrespective of the presence of extrahepatic disease.^102^

Distinguishing HEHE from hepatic angiosarcoma, which is considered a contraindication to LT, can sometimes be difficult based on histology alone.^103^ However, about 90% of HEHE harbour a gene fusion of *WWTR1* and *CAMTA1,* resulting from a translocation between chromosomes one and three. This gene fusion is pathognomonic for HEHE and can be detected by fluorescent *in situ* hybridisation, reverse-transcription PCR, or immunohistochemistry.^104^

In 2017, Lai *et al.* confirmed the role of LT in the management of HEHE while also developing a risk score (HEHE-LT) to stratify patients into low-, intermediate-, and high-risk groups according to their post-LT recurrence risk (Table 5).^105^ In accordance with previous studies,^106^ confined extrahepatic disease was not found to be associated with worse outcomes and should not be a formal contraindication to LT.^105^ However, neoadjuvant systemic therapy is recommended for patients with extrahepatic disease, as is a mandatory waiting period of 120 days before undergoing LT in order to allow for better interpretation of tumour biology. Pulmonary involvement is the most common extrahepatic manifestation.^106^ Combined as well as serial liver and lung transplantation have been performed to curatively treat HEHE with extrahepatic pulmonary disease.^107^ Patients at high risk of recurrence should undergo immunosuppressive tailoring and should be offered adjuvant therapy following LT.^105^

**Table 5:** HEHE-LT score criteria.

Predictor	Points
Waiting time from waitlist registration to LT	
>120 days	0
≤120 days	2
Macrovascular invasion	
Absent	0
Present	5
Hilar LN invasion	
No	0
Yes	3

Modified from Lai *et al.*^105^

HEHE, hepatic epithelioid hemangioendothelioma; LN, lymph node; LT, liver transplantation; 0-2 points, low risk; 3-5 points, intermediate risk; 6-10 points, high risk.

### Colorectal liver metastases

Colorectal cancer (CRC) ranks among the most common malignancies in the Western world and is the fourth most common cause of cancer-related death.^108^^,^^109^ While outcomes for localised disease have improved, outcomes for metastasised disease have remained poor.^109^ Between 25% to 30% of patients with CRC develop liver metastases.^110^^,^^111^ Approximately 17% of patients have synchronous liver metastases at the time of their CRC diagnosis, with 10% of patients developing metachronous liver metastases later on.^110^^,^^112^

For these patients, surgical resection offers the only chance for long-term survival with 5-year OS rates approaching 60%.^110^^,^^113^^,^^114^ This is in stark contrast to patients undergoing palliative treatment who can expect 5-year OS rates of less than 5%.^110^^,^^111^^,^^114^^,^^115^ Moreover, CRLMs are only amenable to resection in about one-quarter of patients, leaving most patients with a bleak prognosis.^110^^,^^111^

Due to the high number of available liver grafts in Norway, the Oslo group embarked on a prospective pilot study to investigate the potential for long-term survival in patients with either synchronous or metachronous non-resectable CRLMs treated with LT (SECA-I).^116^ Patients who had undergone radical excision of the primary tumour with good performance status (ECOG score 0 or 1) and had received a minimum of 6 weeks of chemotherapy were included in the study. Overall, 25 patients were found to be eligible and were included in the study. The dropout rate was 16%. In the end, 21 patients underwent LT. One-year disease-free survival and 5-year OS rates were 35% and 60% respectively, outperforming even the best available systemic treatment.^117^ The recurrence pattern was distinct from patients undergoing liver resection as most recurrences (68%) following LT affected the lungs and not the liver. Of note, more than one-third of patients with pulmonary metastases were eligible to undergo curative resection of their metastases. Furthermore, pulmonary metastases showed slow growth kinetics even in the presence of immunosuppression and their presence did not appear to negatively influence survival.^110^^,^^118^ Again, similarly to HCC (recurrence), considering the advances in current curative therapies and their questionable clinical impact, the use of disease-free survival as an outcome parameter needs to be challenged in future study designs.

Based on the results from the SECA-I trial several risk factors associated with unfavourable outcomes were identified and subsequently integrated into the so-called Oslo score^119^ (Table 6).

**Table 6:** Oslo score with corresponding risk groups.

Predictor (0–4 points)	Recurrence
Size of largest tumour >5.5 cm CEA >80 μg/L Resection of primary to LT <2 years Progressive disease at time of LT	0–2 points: low risk 2–4 points: high risk

Modified from Line *et al.*^119^

CEA, carcinoembryonic antigen; LT, liver transplantation.

While LT for CRLM is not a new idea, the encouraging results from the SECA-I trial gave new life to the concept. The earliest series investigating LT for CRLM stem from the ELTR (European Liver Transplant Registry),^120^ the University of Cincinnati^121^ and the University of Vienna.^16^^,^^122^ The historic 5-year OS rates in these series ranged from 12% to 21% which led many to consider CRLM a contraindication to LT until recently.^120^^,^^123^ Building on the results from the SECA-I trial, the Oslo group followed up with the SECA-II trial, pursuing a more stringent selection policy which led to even better outcomes, resulting in a 5-year OS rate of more than 80%.^124^ Long-term observations from the Oslo group, recently published in JAMA Surgery, demonstrated that selected patients with favourable pretransplant prognostic scoring can achieve long-term survival rates comparable to those for conventional LT indications.^125^

More recently, it was suggested that even in patients with resectable CRLMs, LT could lead to better outcomes than liver resection in patients with a high tumour burden score and low Oslo score.^126^^,^^127^

In 2021, an international consensus guideline on LT for CRLMs was published.^128^ The guideline has distilled the available evidence with the emphasis being on dynamic selection parameters as well as assessment of tumour biology (Table 7). To consider a patient with CRLM for LT, the criteria mentioned in Table 7 with respect to (1) the primary tumour, (2) liver metastases, (3) testing of biology and (4) molecular criteria should be fulfilled. Furthermore, while not specifically addressed in these IHBPA (International Hepato-Pancreato-Biliary Association) guidelines, patients with right-sided primaries should not be listed for LT as outcomes for these patients are poor. No patient with a right-sided primary included in the SECA-I trial was alive at 5-year follow-up.^129^

**Table 7:** Summary of the IHPBA consensus recommendations.

Primary tumour	Liver metastases	Testing of biology	Molecular criteria
• The primary tumour should be resected first according to standard oncologic principles with clear margins (R0) • Patients with primary histology of undifferentiated adenocarcinoma or signet ring cell carcinoma should be excluded • N2 status of the primary is a relative contraindication to LT • Extrahepatic disease must be excluded	• Liver metastases should be technically unresectable as defined by a multidisciplinary tumour board • MTV and TLG could be evaluated for the assessment of tumour metabolic activity when a PET-CT scan is available • Patients with a pretransplant MTV of >70 cc and TLG of >260 g should be excluded	• Patients should have had least one line of FU-based, oxaliplatin-based, or irinotecan-based chemotherapy as part of a bridge to transplantation therapy • The response to bridging therapy should be observed for at least 6 months • The interval from diagnosis of unresectable CRLM to LT listing should be at least 1 year • Chun criteria should be used to assess the treatment response, as RECIST criteria underestimate the response to therapies that have a cytostatic rather that cytotoxic mechanism of action • The development of progressive disease after more than three lines of chemotherapy reflects aggressive biology outside of what would be acceptable to consider LT	• Patients with BRAF V600E-mutated metastatic colorectal cancer should be excluded • Considering the good response rates to immunotherapy in MSI high, MMR deficient patients these patients should be excluded

CRLMs, colorectal liver metastases; FU, fluorouracil; IHPBA, International Hepato-Pancreato Biliary Association; LT, liver transplantation; MMR, mismatch repair; MSI, microsatellite instability; MTV, metabolic tumour volume; PET-CT, positron emission tomography – computed tomography; TLG, total lesion glycolysis.

At present, there are ongoing studies to further define the role of LT in the context of CRLMs. In Toronto (NCT02864485) and Wisconsin (NCT05175092), single-arm, prospective studies are currently underway to explore the utility of LDLT for unresectable CRLMs. In France, a randomised-controlled trial (the TRANSMET study) is comparing LT following standard chemotherapy with standard chemotherapy alone for unresectable CRLMs (NCT02597348). Sweden also has an ongoing randomised-controlled trial (the SOULMATE study) comparing LT plus best-established treatment with best-established treatment alone (NCT04161092).

### Metastatic neuroendocrine tumours

The management of neuroendocrine liver metastases (NELMs) involves different curative and palliative strategies such as surgical resection, peptide receptor radionuclide therapy, transarterial or percutaneous locoregional interventions and medical therapies.^130^ While liver resection still represents the curative treatment of choice in case of resectability,^131^ LT has garnered increasing attention, especially in liver-limited, unresectable disease.^132^^,^^133^ LT was initially considered a salvage therapy for patients with very advanced disease. However, recent studies have shown that LT can be curative in highly selected cases of unresectable liver-limited disease.[132], [133], [134], [135], [136], [137], [138], [139]

LT selection criteria are primarily based on single- and multicentric retrospective cohort studies. In 2007 Mazzaferro *et al.* published the Milan-NET selection criteria which have been modified and adopted by UNOS (Table 8).^140^^,^^141^ The process of selecting patients with NELM for LT relies on the use of high-quality imaging. Contrast-enhanced CT (with arterial phase) is compulsory since NELMs are hyper-vascularised tumours.^142^ Diffusion-weighted MRI should also be part of the diagnostic work-up due to its high specificity, especially in small tumours <1 cm.^143^ ^68^Ga-DOTA PET-CT is the radiologic-diagnostic gold standard as it exhibits both high sensitivity and specificity (82%-100% and 67%-100%, respectively), while also reliably identifying extrahepatic lesions.^144^

**Table 8:** Selection criteria for LT in patients with NELM.

Milan-NET criteria	UNOS/OPTN MELD exception criteria
• Low grade (G1 or G2) NET confirmed by histology • Primary tumour drained by the portal venous system • Primary tumour with all extrahepatic deposits resected with curative intent prior to LT • Non-resectable liver metastases involving up to but not more than 50% of the liver volume • Stable disease/response to therapy for at least 6 months prior to transplant consideration • Age <60 (relative criteria)	• Gastro-entero-pancreatic (GEP) NET with portal venous drainage • G1 or G2 grading following the WHO classification • Bilobar, NELM limited to the liver, not amenable to resection • Tumour metastatic replacement should not exceed 50% of the total liver volume • Resection of primary malignancy and extrahepatic disease without any evidence of recurrence at least six months prior to MELD exception request • Negative metastatic work-up (PET scan, somatostatin receptor scintigraphy) • Tumours in the liver should meet the following radiographic characteristics on either CT or MRI: • **CT scan:** ○ Triple phase contrast lesions may be seen on only one of the three phases ○ Arterial phase: may demonstrate a strong enhancement ○ Large lesions can become necrotic/calcified • **MRI appearance:** ○ Liver metastasis are hypodense on T1 and hypervascular in T2 wave images ○ Diffusion restriction ○ Majority of lesions are hypervascular on arterial phase with washout during portal venous phase ○ Hepatobiliary phase post gadoxetate disodium (Eovist): Hypointense lesions are characteristics of NET

LT, liver transplantation; NELMs, neuroendocrine liver metastases; NET, neuroendocrine tumour; OPTN, Organ Procurement & Transplantation Network; UNOS, United Network for Organ Sharing; WHO, World Health Organization.

Outcomes following LT for NELMs vary widely, with 5-year OS rates ranging from 36% to 97%.^133^^,^^138^^,^[145], [146], [147], [148], [149], [150], [151], [152], [153] However, a more homogenous picture appears when considering only patients fulfilling the Milan-NET criteria. The Mazzaferro group described 10-year OS rates between 79.6% and 93%, establishing the superiority of LT compared to conservative treatment (22.4%) or liver resection (75%).^133^^,^^138^^,^^154^ Similarly, a recent large multicentre, retrospective study showed that LT for NELMs resulted in a survival benefit compared to liver resection (median OS 197 months *vs*. 119 months and 5-year survival 73% *vs.* 52.8%), again, exclusively considering patients within the Milan-NET criteria and with low grade tumours (Ki-67 ≤5%).^155^

Similar to other entities, dynamic selection criteria with a stronger emphasis placed on tumour biology are desirable since the current stringent criteria might exclude patients (*e.g.* patients with higher tumour burden and higher grading) who could potentially benefit from LT.^152^

## Ethical and technical aspects of transplant oncology

Despite LT having proven to be the best treatment option for prespecified oncologic indications it is not immediately available to all patients in need due to the limited supply of donor organs. With patients on the waiting list competing for organs, one could argue that outcomes following LT for oncologic indications should be comparable to outcomes for non-oncologic ones.^119^ Over the last few decades outcomes for LT have generally improved with 5-year OS rates increasing from 51% in the late 1980s to 73% currently.^156^ However, the most striking improvements were observed for oncologic indications with 5-year OS rates now reaching 70%, compared to 23% in the late 1980s. Still, designing exceptional MELD point criteria needs to be done sensibly to avoid over-prioritisation of patients with oncologic indications while maintaining acceptable outcomes. Ultimately, as new evidence emerges, allocation policies for patients with hepatic malignancies will need to be adjusted to guarantee fair access to liver grafts for all patients. Therefore, strategies to expand the donor pool are urgently needed if we want to be able to offer patients the best available treatment options. Relying on liver grafts from extended criteria donors (ECDs) and donation after circulatory death (DCD) donors is one such strategy to expand the donor pool. However, the use of ECD and DCD organs comes with a higher risk of developing primary non-function, early allograft dysfunction as well as post-transplant cholangiopathy.^157^ With the clinical implementation of liver machine perfusion strategies, a platform for organ reconditioning and viability assessment has become available.[158], [159], [160] The VITTAL study, utilising nationally declined liver grafts, has shown that the application of normothermic machine perfusion (NMP) permits selection of grafts suitable for transplantation, thereby safely expanding the donor pool.^161^ Furthermore, concepts such as dual hypothermic oxygenated machine perfusion (D-HOPE) followed by controlled rewarming (COR) and NMP (D-HOPE-COR-NMP) allow for a period of graft reconditioning (during D-HOPE) followed by the possibility of viability assessment (during NMP).^162^^,^^163^ The available data clearly positions liver machine perfusion at the forefront of strategies aimed at addressing organ scarcity. Liver machine perfusion enables mitigation of the increased risk incurred through the utilisation of ECD and DCD organs by enabling reconditioning as well as better selection of grafts suitable for transplantation.

While machine perfusion may provide some relief in terms of organ shortages, other options to expand the donor pool – such as SLT and LDLT – need to be explored.

Reduced size transplantation was first reported by Bismuth *et al.* in 1984, who transplanted a reduced size left-lateral graft into a paediatric recipient.^164^ The remaining segments (IV-VIII) were not transplanted which had a negative effect on the adult organ pool. Pichlmayr *et al.* were the first to transplant one donor liver into two recipients (SLT) following *ex situ* splitting, thereby increasing the donor pool but only for paediatric recipients.^165^ Later Broering *et al.* reported successful full-left full-right *in situ* splitting where both grafts were transplanted into two adult recipients. Although full-left full-right splitting has continued to evolve and has now reached technical adulthood with outcomes similar to those of whole LT,^166^ it has not gained widespread acceptance and, like LDLT, is currently massively underutilised.

In Western countries, LDLT accounts for only 5% of the overall LT volume.^156^^,^^167^ Past reports of inferior outcomes as well as concerns regarding donor morbidity and mortality have hampered more widespread implementation of LDLT in the Western hemisphere.[168], [169], [170], [171], [172] However, more recent data have shown that LDLT has become a relatively safe procedure for the donor. Donor morbidity (Clavien-Dindo ≥III) is approximately 10% in experienced centres.^173^^,^^174^ More importantly, not a single donor death has been reported in recent single-, multicentre and registry studies.[173], [174], [175], [176], [177], [178], [179] LDLT is a highly technical procedure requiring a certain expertise to achieve excellent outcomes.^180^^,^^181^ With increasing experience, single-centre studies, national data analysis as well as a recent meta-analysis have demonstrated equivalent or superior outcomes following LDLT compared to deceased donor liver transplantation (DDLT).^173^^,^^182^^,^^183^ For patients with HCC, having the possibility to undergo a LDLT significantly decreases the risk of death in intention-to-treat analyses.[184], [185], [186] While early reports raised concerns regarding higher HCC recurrence rates following LDLT,^187^^,^^188^ recent studies have demonstrated that post-transplant (as-treated) survival and tumour recurrence are similar for LDLT and DDLT recipients despite the fact that patients undergoing LDLT more often have a higher tumour burden outside of acceptable transplant listing criteria.^184^^,^^186^ The intention-to-treat survival benefit obtained from LDLT is probably a reflection of the shorter waiting time combined with a decrease in waitlist dropout.

LDLT has also been successfully used in the context of other oncologic indications.[189], [190], [191] In 2022, Hernandez-Alejandro *et al.* published the results of the first prospective multicentre study investigating LDLT for unresectable CRLM.^189^ The results were promising with Kaplan-Meier-estimated survival of 100% at 1.5 years. However, the follow-up was short and recurrence rates were high (30%). Therefore, the long-term oncologic results remain to be elucidated.

**Fig. 2:**
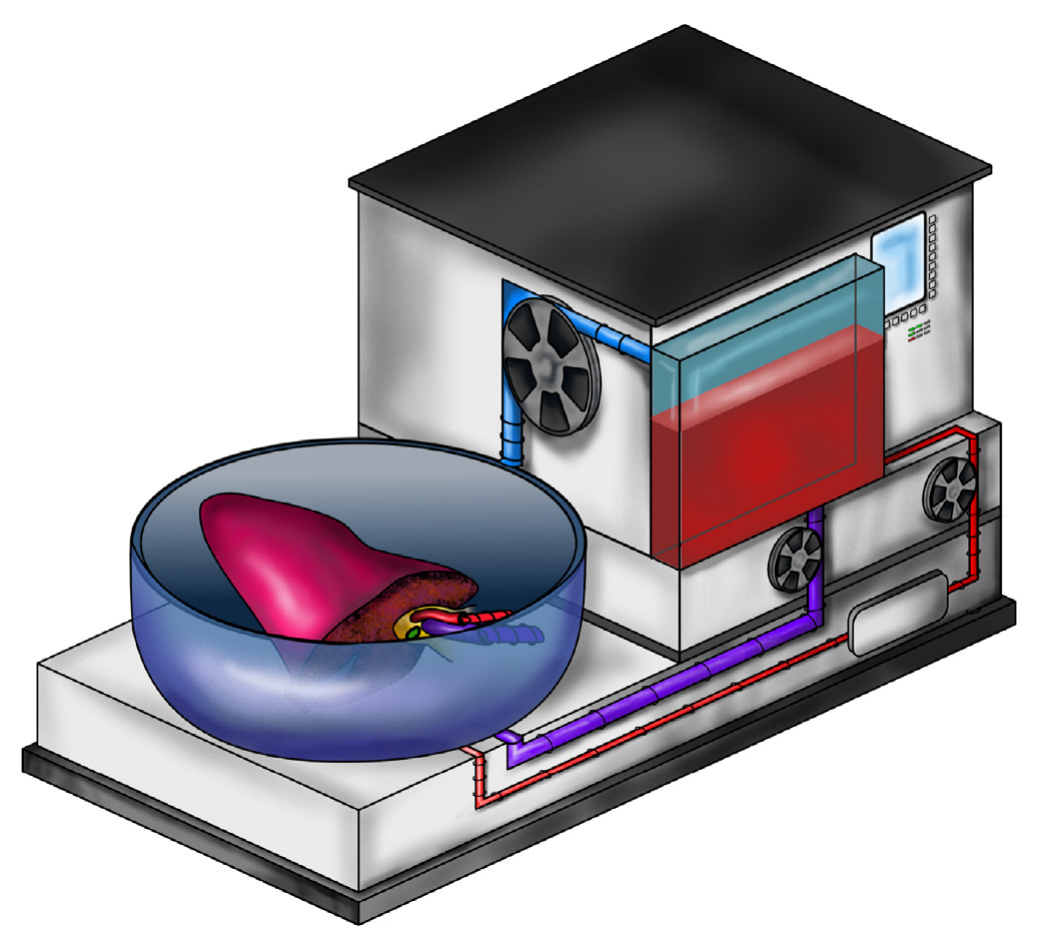
Combining technical innovations such as machine perfusion with innovative surgical concepts such as RAPID has the potential to further bridge the gap between organ supply and demand.

When considering LDLT, the transplant benefit (*i.e*. the life years gained with transplantation compared to remaining on the waitlist) should be high to justify the perioperative risks for the living donor. This seems to be the case for selected oncologic indications where LT leads to 5-year survival rates upwards of 60% while the alternative treatment offers 5-year survival rates of 10% or less.^192^ Within this context, LDLT lends a different perspective to LT as (1) LDLT is not limited by restrictions imposed by the nationwide allocation systems,^193^ (2) the graft from a living donor is a private donation, meaning it is only intended for a specific recipient, while the graft from a deceased donor is considered to be a public donation^194^ and thus (3) LDLT does not interfere with the deceased donor pool. Moreover, LDLT not only does not interfere with the deceased donor pool but actually benefits all patients waitlisted for DDLT as the LDLT recipients remove themselves from the deceased donor waiting list.^195^ The combination of technical innovations with innovative surgical concepts may help to further address this unmet need of transplant oncology (Fig. 2).

The RAPID technique is another strategy to increase the number of available liver grafts for adult recipients with oncologic indications, by splitting livers from deceased donors and subsequently having two grafts, an extended-right and a left-lateral graft, available for transplantation.^196^ The concept being that the recipient hepatectomy becomes a two-stage procedure: following a left- or left-lateral hepatectomy, with care being taken to avoid cutting through the tumour, the recipient receives a left-lateral graft. The insufficient metabolic mass of the left-lateral graft is compensated for by the remaining native liver which is left *in situ* during the first stage of the procedure. After sufficient hypertrophy of the left-lateral graft, the remaining cancerous liver is removed. Hence, the RAPID procedure allows transplantation of an extended-right graft and a hypertrophied left-lateral graft into two normal sized adult recipients. Königsrainer *et al.* described the RAPID procedure in the setting of living donation, where a live donor undergoes resection of the left-lateral segments and termed the concept LD-RAPID.^197^ The LD-RAPID procedure thus minimises the risk for the living donor, by enabling the donation of a smaller liver part, while still providing sufficient liver volume for an adult recipient and is thus in keeping with the ethical principle of double equipoise.^198^

One key aspect of LT is optimising the donor and recipient matching process in order to balance the overall risk of the procedure.^199^ Constellations where a sick patient with severe portal hypertension and a high laboratory MELD score is allocated a marginal liver graft should be avoided. In general, patients with oncologic indications have lower laboratory MELD scores and less portal hypertension compared to patients listed for other indications.^200^ This has implications for the allocation process in the setting of transplant oncology: (1) Grafts from ECDs which would otherwise have been declined for transplantation can be safely utilised in recipients with low laboratory MELD scores, as shown by McMillan *et al.*^79^ Liver machine perfusion strategies may allow these boundaries to be pushed further. (2) When transplanting technical variant grafts in the context of SLT and LDLT, recipients without severe portal hypertension and low laboratory MELD scores require less metabolic mass, *i.e*. the graft-to-recipient weight ratio can be lower than would be required in a recipient with a high laboratory MELD score.^201^ Within this context, technical advances such as LD-RAPID and its variations^202^ have opened the door to significantly extend the role of LDLT, especially in Western societies where LDLT rates lag behind those of Asian countries.^203^

In summary, patients with oncologic indications and low laboratory MELD scores may preferentially be allocated grafts from ECDs without compromising outcomes. While LDLT provides an intention-to-treat survival benefit compared to DDLT, it may also be performed in patients not expected to meet the same post-LT survival thresholds as those transplanted for other indications, provided (1) the transplant benefit is high enough to justify the risk for the living donor and (2) both the donor and recipient have consented and have realistic expectations of the achievable outcomes.^195^ The concept of double equipoise evaluates the relationship between the recipient's need, the donor's risk, and the recipient's outcome. Each donor-recipient pair is considered as a unit. It is the transplant team’s job to analyse whether the specific recipient's benefit justifies the specific donor's risk for a particular oncologic indication and protect donors from donation if the potential harm outweighs the expected benefit.^204^^,^^205^ Currently, no consensus exists about what constitutes acceptable recurrence and donor risks, and ethical considerations may differ in different societal contexts.^195^^,^^205^

## Outlook

With our understanding of tumour biology improving, patient selection in transplant oncology will move away from a static, imaging-based approach towards a more dynamic process. The NYCA score is one crucial stepping-stone along this path. Testing of biology and response to neoadjuvant treatment will play a bigger role when deciding who to list for LT.

The Mayo protocol has been groundbreaking in providing a framework where response to neoadjuvant treatment enabled the selection of patients who obtain the highest transplant benefit. McMillan *et al.* used a similar approach for iCCAs where patients had to undergo neoadjuvant treatment and show favourable response before being considered for LT.^79^ In the context of HCC, locoregional therapies have historically been used to bridge and downstage patients while systemic therapies were reserved for the palliative setting. This might change in the future as cases of LT following successful downstaging using a combination of atezolizumab-bevacizumab have been reported.[206], [207], [208], [209] Schmiderer *et al.*^207^ reported a case where a patient with an advanced HCC (macrovascular invasion, BCLC C) showed remarkable radiologic and biochemical response to treatment with atezolizumab-bevacizumab and subsequently underwent LT. The explant histology confirmed the impressive radiologic response, yielding a RETREAT score of 2 points which, in theory, indicates a low risk of tumour recurrence, keeping in mind that the RETREAT score has not been validated in this setting. Currently, there is an ongoing study with the goal to evaluate the safety and feasibility of pretransplant treatment with atezolizumab-bevacizumab for patients outside the Milan criteria (NCT05185505).

Pretransplant use of an immune checkpoint inhibitor (ICI) was first reported by Nordness *et al.*^210^ A patient with a HCC received treatment with nivolumab, a programmed death 1 (PD-1) inhibitor, before LT. In the immediate postoperative period, the patient developed acute hepatic necrosis due to a profound immune reaction and died. Similarly, Chen *et al.*^211^ reported a case of fatal acute hepatic necrosis following the pretransplant use of toripalimab, which, like nivolumab, is an anti-PD-1-antibody. Tabriazian *et al.*^212^ published a series on nine patients receiving nivolumab before LT. One patient developed mild acute rejection associated with low tacrolimus levels, apart from that, no severe rejection episodes occurred and no patient experienced graft loss. Graft rejection is the main concern with peri-transplant ICI use and the PD-1/programmed death ligand 1 (PD-L1) pathway has been reported to be critical for graft acceptance.^213^^,^^214^ Therefore, some authors have suggested the need for a minimum time period between the last ICI dose and LT (*i.e*., a washout period) to minimise the risk of rejection.^215^ The half-life of nivolumab and atezolizumab is 27 days and plasma levels typically decline below significant levels after three half-lives, which is why a washout period of 3 months has been suggested.^216^ However, reports in the literature are conflicting and the existing evidence mainly consists of case reports and small case series reporting on a heterogenous cohort of patients.^216^ In the series reported by Tabrizian *et al.*, four of nine patients received their last dose of nivolumab within 14 days of LT, and none of these patients showed any signs of rejection.^212^ In the reports by Nordness and Chen *et al.*, the recipients received their last ICI dose (nivolumab and toripalimab) 8 and 93 days before LT, respectively, and both recipients developed fatal acute hepatic necrosis.^210^^,^^211^ Thus, other factors, apart from the time since the last ICI dose, seem to be at play here and serum half-lives alone might not be a reliable indicator when determining the minimum required washout period.^217^ Receptor occupancy and the effect on T-cell activity has reportedly exceeded the half-lives of the respective ICIs significantly.^217^^,^^218^ This is supported by the observation that drug-related adverse events and anti-tumour effects have been observed months after drug administration.^219^

An analysis of 43 patients who have received ICIs before LT showed that eight patients (18.6%) developed severe graft rejection and four of those patients (9.3%) died.^218^ In the post-transplant setting the graft rejection rate among LT recipients receiving ICIs was 28.8% (15 out of 52). Seven patients (13.5%) died due to liver failure related to graft rejection.^220^ Compared to other solid organs, the liver is immunologically privileged^221^ and graft failure due to acute rejection is extremely rare among recipients who have not received ICIs.^222^

Overall, post-LT use of ICIs seems to be riskier than pre-LT use. Therefore, it is essential to carefully assess potential risks and benefits when selecting LT recipients for ICI treatment. Furthermore, if ICI treatment is initiated, recipients should undergo close monitoring.^223^ Montano-Loza *et al.* recently published a decision table for the use of ICIs in LT recipients according to the individual immunological risk and oncological benefit.^223^

Interestingly, despite the overall limited experience with atezolizumab, a PD-L1 antibody, in the peri-transplant setting, none of the ten patients (five pre- and five post-LT) reported to have received atezolizumab developed graft rejection.^208^^,^^209^^,^^218^^,^^224^ Whether peri-transplant use of atezolizumab is safer than the use of other ICIs needs to be further investigated.^224^ In terms of patient selection, graft PD-L1 expression has been discussed as a potential biomarker as it has been suggested to correlate with the risk of rejection.^218^^,^^220^ In the post-transplant setting, four out of nine recipients for whom graft biopsy was available had positive PD-L1 staining and all four experienced graft rejection. In the other five recipients with negative PD-L1 expression no rejection was observed.^220^ While PD-L1 expression might be a useful parameter when selecting LT recipients for ICIs, its predictive value in the pretransplant setting remains unclear. In both cases reported by Nordness *et al.*^210^ and Chen *et al.*^211^ pretransplant PD-L1 staining was negative while post-transplant PD-L1 expression was positive, indicating that PD-L1 expression might be the graft’s attempt to escape the recipient’s immune response. Blocking that escape mechanism may cause graft rejection.^218^ All in all, a lot of questions remain unanswered and more studies, to elucidate mechanisms, risk factors and biomarkers of ICI-mediated rejection are required to establish safe protocols for the pre- and post-transplant use of ICIs. In light of a washout period potentially being crucial when it comes to pretransplant ICI use, another point can be made in favour of LDLT, which, compared to DDLT, is a planned, scheduled operation that allows for optimal timing and coordination of neoadjuvant treatment regimens with the transplant procedure.

Besides rejection, another post-transplant concern specific to patients undergoing LT for primary or secondary hepatic malignancies is tumour recurrence. Beyond the overall minimisation of immunosuppression, which includes tapering steroids and eventually withdrawing them,^225^ early introduction of an mTOR inhibitor in conjunction with a calcineurin inhibitor taper or switching entirely to an mTOR inhibitor have been recommended to reduce the risk of tumour recurrence.^128^^,^^226^

**Fig. 3:**
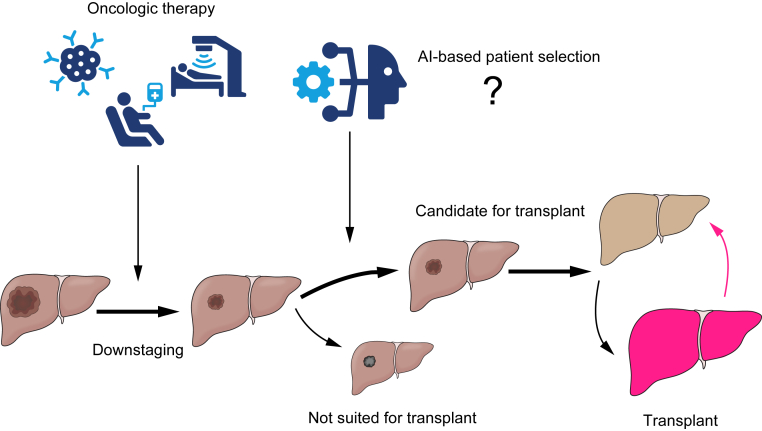
Summary of current and potential future concepts of transplant oncology.

Provided the problem of organ scarcity can be successfully addressed, the multidisciplinary approach to transplant oncology, combining new systemic treatments with LT, offers a path to provide selected patients, who would have previously received palliative care, with a potentially curative treatment option (Fig. 3).

## Financial support

The authors did not receive any financial support to produce this manuscript.

## Authors’ contributions

Conceptualization and drafting FJK, RO, MM; drafting and critical revision RB; critical revision FJK, GS, BS, HT, SS, CM, SS, RO, MM; Final approval: all authors.

## Conflicts of interest

The authors of this study declare that they do not have any conflict of interest.

Please refer to the accompanying ICMJE disclosure forms for further details.
